# Key plasma microRNAs variations in patients with *Plasmodium vivax* malaria in Iran

**DOI:** 10.1016/j.heliyon.2022.e09018

**Published:** 2022-02-27

**Authors:** Ramtin Hadighi, Aliehsan Heidari, Parviz Fallah, Hossein Keshavarz, Ziba Tavakoli, sholeh Mansouri, Mehrdokht Sadrkhanloo

**Affiliations:** a Department of Parasitology and Mycology, Iran University of Medical Sciences, Tehran, Iran; b Department of Parasitology, School of Medicine, Alborz University of Medical Sciences, Karaj, Iran; c Department of Medical Laboratory Sciences, Para-Medicine Faculty, Alborz University of Medical Sciences, Karaj, Iran; d Department of Medical Parasitology and Mycology, School of Public Health, Tehran University of Medical Sciences, Tehran, Iran; e Tehran Medical Branch, Islamic Azad University, Tehran, Iran

**Keywords:** MicroRNA, Malaria, *Plasmodium vivax*, Stem- loop RT-PCR

## Abstract

**Introduction:**

As the cause of RBC infection and splenomegaly, malaria remains a major parasitic disease in the world. New specific biomarkers such as MicroRNAs (miRNAs) are developed to accurately diagnose malaria and clarify its pathologic changes. This study aimed at evaluating changes in the plasma miRNAs markers of *Plasmodium vivax* in patients with malaria in Chabahar, Iran.

**Materials and methods:**

For the present descriptive-analytical study conducted in 2018, we collected blood samples from 20 individuals. Real-time quantitative Polymerase Chain Reaction (RT-qPCR) was used to measure the plasma levels of miR-145, miR-155, miR-191 and miR-223-3p.

**Results:**

The 2-ΔΔCT method of Real-time PCR showed the plasma levels of miR-223, miR-145 and miR-155 to respectively be 5.6, 16.9 and 1.7 times higher in patients with *P. vivax* compared to those in healthy individuals. The expressions of all the three miRNAs significantly increased in patients with malaria compared to in the controls (P < 0.05). The expression of miR-191 was 1.405 times higher in patients with malaria compared to that in the controls, although the difference was statistically insignificant.

**Conclusion:**

The present study found *P. vivax* to change host miRNAs such as miR-223, miR-145 and miR-155. These small molecules thus appeared to constitute biomarkers for *P. vivax* malaria assessment.

## 1 Introduction

With approximately 219 million infected cases and 409000 deaths in 2016, malaria constitutes a globally major parasitic disease [1]. *P. vivax*, *P. falciparum*, *P. malariae*, *P. ovale* and *P. Knowlesi* as the species of *Plasmodium* cause human infections [2].

As a health threat, *P. vivax* contributes to almost 90% of malaria cases in the south and southeast of Iran [3]. The importance of *P. vivax* malaria lies in the fact that it may relapse after weeks to months [4, 5]. Giemsa stained thin and thick blood smears are commonly used to diagnose malaria. The low sensitivity and specificity of this test, false-negative results and inaccurate identification of *Plasmodium* species in patients with low-density malaria [6, 7] delay the treatment of malaria and increase the risk of complications in case two species of *Plasmodium* cause the infection.

Modern diagnostic methods, including malaria antigen isolation, flow cytometry, antibody isolation, and specific biomarkers such as MicroRNAs (miRNAs) were developed to more effectively diagnose malaria. Furthermore, mirRNAs are effective in clarifying the pathological changes of malaria. As small non-coding RNAs, miRNAs control the expression of genes related to cell growth, differentiation and death by inhibiting target genes on mRNA [8]. The miRNA expression depends on the type of tissue and pathophysiological conditions [9]. The expression of miR-451 is specifically observed in the circulating erythroid line [10], but its expression in red blood cells is not related to the proliferation of *Plasmodium* in red blood cells [11].

In recent years, cases of cerebral malaria or severe malaria with pathological complications caused by Plasmodium *vivax* mono-infection have been reported in the endemic areas of India and Brazil [12, 13, 14]. Acute respiratory distress syndrome was also reported in a 60-year-old patient with *Plasmodium vivax* [15]. MicroRNAs can be used to differentiate severe from uncomplicated malaria caused by *Plasmodium falciparum*. The level of some microRNAs has been changed due to pathological changes in malaria [16].

*Plasmodium falciparum* increased the plasma levels of hsa-miR-150-5p and hsa-miR-3158-3p in patients with fatal cerebral malaria compared to those in patients with non-fatal malaria [17]. MiR-155-5p and MiR-223 were substantially dysregulated in mice groups with cerebral malaria as compared with mice that were not infected with malaria [18]. *P. vivax* can cause severe malaria like *P. falciparum*, and can be sequestered in the spleen, especially in asymptomatic cases; therefore, microRNA can be used to distinguish such severe and asymptomatic cases [19, 20].

Hence, MicroRNA can be used to diagnose these conditions of malaria.

Extracellular miRNAs are highly stable in plasma. Given the changes in plasma miRNAs in many diseases, including malaria, they are considered as non-invasive biomarkers to track their pathological effects [21].

Among the several miRNAs in peripheral blood mononuclear cells of individuals with HIV/AIDS, miR-223 and miR-191 are downregulated during HIV/AIDS infection. Accordingly, these miRNAs can be used as biomarkers to detect HIV infection [21].

The infections with *Toxoplasma gondii* and *Leishmania major* upregulate miR-155 in host cells. Negative correlations were observed between miR-145-5p and the peak parasitemia in the hearts of mice 30 days after their infection with *Trypanosome cruzi* [22]. *P. chabaudi* infections induce an organ-specific response of the miRNA expression. MiR-145 is downregulated by 0.48–0.14-fold in both the spleen and liver of female C57BL/6 mice infected with *P. chabaudi* [23].

Berillo et al. reported decreased circulating let-7g-5p and miR-191-5p as independent biomarkers of chronic kidney disease among patients with hypertension, which could have pathophysiological and therapeutic implications [24].

The regulatory role of miRNAs in the pathogenesis of severe malaria was reported in 2018 [25]. The plasma levels of miR-451 and miR-16 were found to decrease in patients with malaria [26]. Moreover, the role of miRNAs and gene expression in different diseases such as malaria has been well addressed in literature. Changes in the plasma levels of miRNAs can help diagnose severe malaria in patients with *P. vivax*. This study aimed at evaluating changes in the plasma miRNAs of *P. vivax* in patients with malaria in Chabahar, Iran.

## 2 Materials and methods

### 2.1 Sampling

Malaria in Iran mainly occurs in Sistan and Baluchestan province, which borders Pakistan and Afghanistan [2].

The present descriptive-analytical study collected blood samples from 20 individual including 10 patients with malaria caused by *P. vivax* presenting to Chabahar rural health centers in Sistan and Baluchestan province and 10 healthy individuals as the control group. This research was approved by Biomedical Research Ethics Committee of Iran University of Medical Sciences (IR.IUMS.FMD.REC.1397.006). Informed consent was obtained from all patients/participants in the current study.

The plasma of 2 ml of venous blood taken from each individual in EDTA tubes was separated through centrifugation.

Two malaria experts confirmed the presence of *P. vivax* by examining patients’ peripheral blood smears. The whole blood sample underwent DNA extraction for *Plasmodium* gene analysis and plasma was used for human miRNAs detection.

Thick and thin film blood smears were prepared and stained with 3% Giemsa. The parasite density in thick blood films was calculated as the number of parasites per mm^3^ of blood by multiplying the number of asexual parasites per 200 WBCs by 40.

Nested PCR using *Plasmodium* 18 subunit ribosomal ribonucleic genes and specific primers was applied to identify Plasmodium species and mixed-species infections, and to confirm infection with *P. vivax* in patients with malaria. The sequences of the primers and detailed nested PCR amplifying methods have been previously elucidated [27]. Both positive and negative controls were used to ensure the accuracy of nested PCR. PCR products were electrophoresed on 2% agarose gel and stained with DNA Safe Stain to observe bands under an ultraviolet transilluminator. Nested PCR was considered positive when a ∼120 bp band was illustrated for *P. vivax* [28]. Besides nested PCR for detecting *P. vivax* genetic marker merozoite surface protein 5 (MSP5) was also used to identify and genotyping *P. vivax* samples. MSP5 Exon1 was amplified using PCR and PCR products were sequenced to find specific sequences for *P. vivax*. Table 1 presents the sequences of the primers used for *P. vivax* identification and Pvmsp5 recognition.

**Table 1:** The sequences of the primers used for nested PCR and Pvmsp5 analyses.

Primers	Specificity	Sequence
rPLU 5	Genus of *Plasmodium*	CCTGTTGTTGCCTTAAACTTC
FrPLU 6	Genus of *Plasmodium*	TTAAAATTGTTGCAGTTAAAACG
rVIV 1	*Plasmodium vivax*	CGCTTCTAGCTTAATCCACATAACTGATAC
rVIV 2	*Plasmodium vivax*	ACTTCCAAGCCGAAGCAAAGAAAGTCCTTA
Pvmsp-5 F	Exon1	CGCGTCGTGTTAGCTATCCA
Pvmsp-5 R	Exon1	CATCGTCTGCCTTGTGTTCG

This study included 10 patients with malaria infected only with *P. vivax*. All the patients underwent Wright, Widal and RF tests, and were asked about their comorbidities. No other diseases were therefore reported in the patients. RT-qPCR was performed to measure the expression levels of miR-145, miR-155, miR-191 and miR-223-3p.

### 2.2 Predicting and selecting miRNA

**Figure 1:**
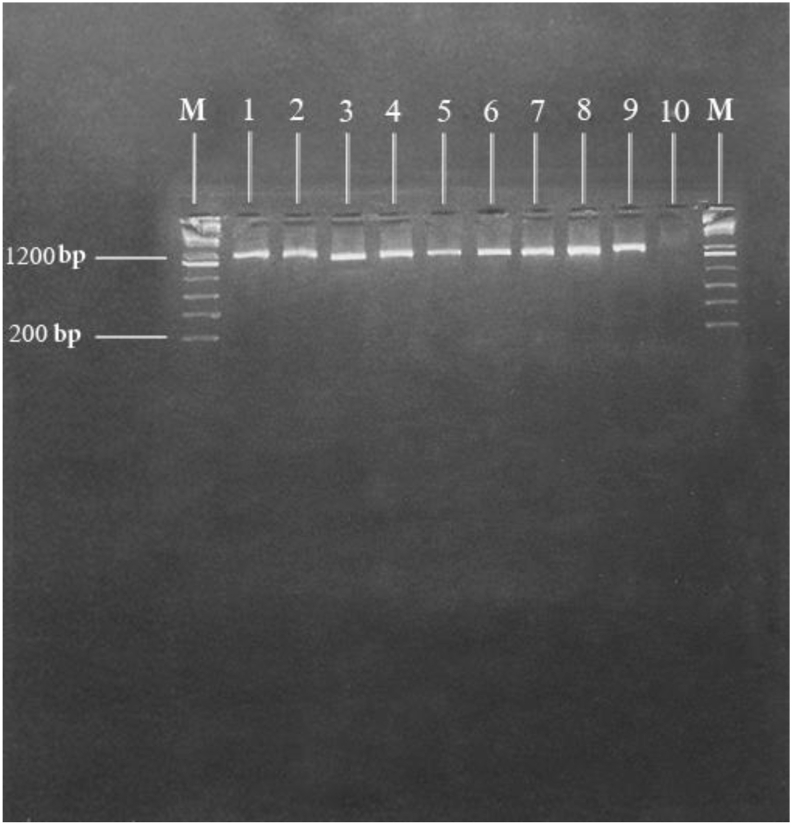
Gel electrophoresis of stage 1 of nested PCR product obtained from P.vivax positive samples in southeast Iran. The DNA size markers are a 200 bp ladder shown on the left and right. Lane1 to9 are Patients Plasmodium vivax isolates. Lane 10 is negative control.

According to the literature, miRNAs were selected using miRNA prediction software packages, including TargetScan (http://www.targetscan.org)(Version 5.1), PicTar, DIANA microT and miRands [15, 17, 18, 19]. Four miRNAs were selected based on their scores in KEGG (https://www.genome.jp/pathway/map05144) and DAVID (http://david.abcc.ncifcrf.gov/) to check their signaling pathways. miRNAs might play a role in RBCs and *Plasmodium* infection (Supplementary Table 1 and Figure 1).

### 2.3 Designing the primers and stem-loop

The sequences of 18 potentially-interfering miRNAs were retrieved from the Sanger Center miRNA Registry (http://www.sanger.ac.uk). This study investigated human miRNAs, including hsa-miR- 155, 145, 191 and 223. To increase the flexibility and length of the stem-loop structure and design its internal universal reverse primer and achieve the necessary sensitivity, the sequence reported by Chen et al. [29] was modified by adding 14 nucleotides to the original sequence. Substitutions were also made to decrease the melting temperature of the stem part. This newly-designed structure was used to individually detect the miRNAs given that few nucleotides complementary to the 3′UTR of miRNA were added to each stem-loop. An almost complete sequence of each miRNA designed in allele ID6 was used as the forward primer in real-time PCR (Table 2). The secondary structure of the amplicon was analyzed using the ‘‘mfold’’ analysis (http://mfold.rna.albany.edu/q=mfold).

**Table 2:** miRNA accession numbers designed RT stem-loops, and primers.

miRNA	Specific forward Primer	RT specific stem-loop primer
Hsa-miR-155	CCGTTAATGCTAATCGTGA	GTCGTATGCAGAGCAGGGTCCGAGGTATTCGCACTGCATACGACACCCCT
Hsa-miR-145	TGGAAGGTTGAGAACTGA AT	GTCGTATGCAGAGCAGGGTCCGAGGTATTCGCACTGCATACGACACCCCT
Hsa-miR-191	AAGGAATCCCAAAAGCAG	GTCGTATGCAGAGCAGGGTCCGAGGTATTCGCACTGCATACGACTTCGTC
Hsa-miR-223	TGGCTGTCAGTTTGTCTAATAC	GTCGTATGCAGAGCAGGGTCCGAGGTATTCGCACTGCATACGACATTATG
RNAU6	AAGGATGACACGCAAATTC	GTCGTATG AGAGCAGGGTCCGAGGTATTC GCACTGCATACGACAAAAATATGG
**Universal reverse primer:** GAGCAGGGTCCGAGGT		

The specificity of each miRNA-specific primer was verified in BLAST (http://blast.ncbi.nlm.nih.gov/). A specific RT stem-loop primer was used in the presence of all the other miRNA-specific primers to determine the specificity of the experiments for each cDNA synthesized through cross-amplification real-time PCR. The relative expression was analyzed based on the ratio of the target miRNA threshold cycle (C_t_) to the C_t_ of RNAU6 (U6) as the reference gene.

### 2.4 MiRNA extraction and cDNA synthesis

According to the manufacturer's protocol, the MiRNA extracted using QIAZOL RNA (Qiagen, USA) for the case and control groups was reverse transcribed to cDNA using stem-loop RT specific primers (for predicted miRNAs and RNAU6) and M-MuLV Reverse Transcriptase (Thermo Scientific). RNAs and cDNAs were stored at −70 °C and −20 °C, respectively.

### 2.5 Level of microRNA expression based on real-time PCR

A two-step Real-Time PCR was performed using Applied Biosystems StepOne and RealQ Plus 2x Master Mix Green (Ampliqon, Denmark) after extracting RNA and constructing cDNA with the RT-stem-loop primer assigned to each miRNA by adding the end portion of the miRNA sequence. The short-length RNAU6 (U6) gene was used as an internal control to normalize the findings (Tables 3 and 4). A No Template Control (NTC) was prepared as the negative control for all the cited materials except for cDNA.

**Table 3:** Materials needed for Real-Time PCR reaction in Applied Biosystems StepOne.

Materials	Concentration (μM)
Master Mix (SYBRR Premix Ex TaqTM- Takara)	1X (12.5) μl
Forward Primer (10pmol)	0.5 μl
Universal Reverse Primer (10pmol)	0.5 μl
Template (cDNA)	2 μl
diH2O	To volume 25 μl

**Table 4:** Schedule of Real-Time qPCR reaction steps for miRNAs.

Stage	Temperature	Time	Cycles
Enzyme activation	95 °C	30 s	1
Denaturation	95 °C	5 s	45
Annealing and Extension	60 °C	30 s	
To draw the Melt curve, increased the temperature from 50 °C to 90 °C			

The 2-ΔCt method was used to compare the plasma expressions of miR-155, miR-145, miR-191, miR-223-3p and RNAU6 between patients with *P. vivax* malaria and healthy individuals.

### 2.6 Statistical analysis

The Mann Whitney U test was used to determine differences in the miRNA expression of miR-145, miR-155, miR-191 and miR-223 between the patients and the controls. The Fisher exact test was used to compare the two groups in terms of gender. The Spearman test was employed to determine correlations between parasitemia and levels of the individual miRNAs. The Spearman correlation and independent t-test were also used to determine correlations between age/gender and levels of the study miRNAs, respectively.

## 3 Results

**Figure 2:**
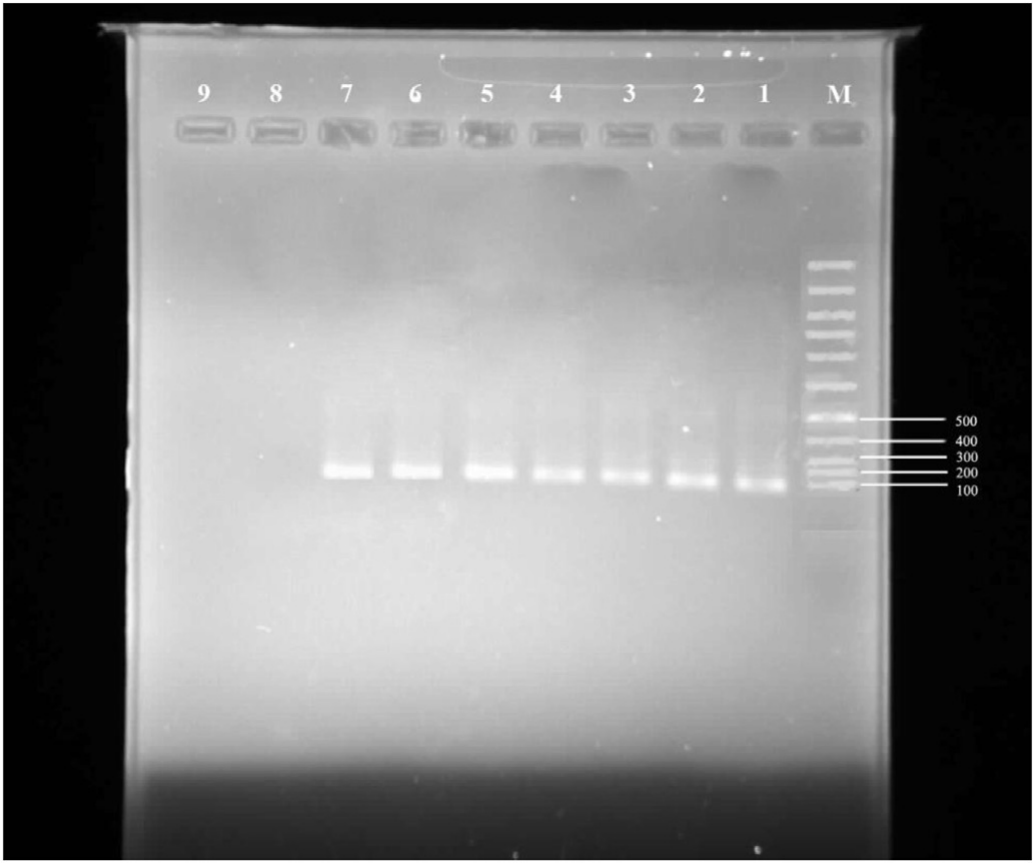
Gel electrophoresis of the nested PCR product obtained from P.vivax positive samples in southeast Iran. The DNA size marker is a 100 bp ladder shown on the right. Lane1 to7 are Patients Plasmodium vivax isolates. Lane 8 and 9 are negative controls.

**Figure 3:**
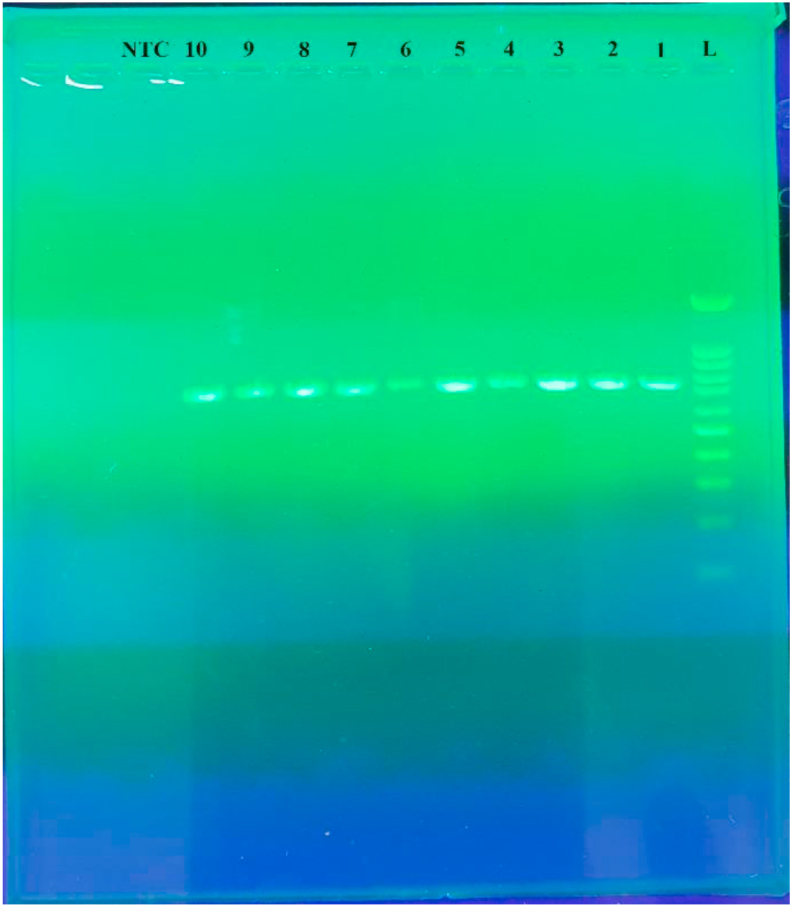
Gel electrophoresis of the PVmsp5 PCR product obtained from P.vivax positive samples in southeast Iran. The DNA size marker is a 100 bp ladder shown on the right. Lane1 to10 are Patients Plasmodium vivax isolates. Lane NTC is negative control.

According to the demographic data, six out of the ten patients with *P. vivax* malaria were male and four were female. The patients were 24–60 years old. The Fisher exact test obtained as 1 suggested no significant differences between the two groups in terms of gender (P > 0.05). Despite the wide age range, insignificant differences were observed between the case and control groups in individual age ranges (Table 5). Gender and age were not significantly related to infection with *P. vivax* (P > 0.05). Out of the ten patients, six (60%) were Afghan, three (30%) Iranian and one (10%) Pakistani. Despite observing chills, fever, sweating, general weakness and mild anemia, no symptoms of complicated *P. vivax* malaria were observed in the study patients. A specific band for *P. vivax* was obtained in all the ten patients with malaria (Figures 1 and 2). Figure 3 shows the gel electrophoresis of the Pvmsp5 PCR product obtained from the positive samples for *P. vivax*.

**Table 5:** Frequency distribution of the study population by age and sex.

Variables	Patients group	Control group	P- value
**Gender**			P > 0.05
Male	6 (60%)	5 (50%)	
Female	4 (40%)	5 (50%)	
**Age**			
<30 Years	3 (30%)	4 (40%)	
30–50 Years	5 (50%)	3 (30%)	P > 0.05
>50 Years	2 (20%)	3 (30%)

Parasitemia was ranged as 1,000–30,000 P/μl of blood. Parasitemia (P/μl) in the ten patients with *P. vivax* was obtained as 23920, 3360, 18800, 1000, 8760, 4280, 23640, 30000, 13280, and 4120. According to the Spearman's rho test, no correlations were observed between parasitemia and levels of the study miRNAs (P > 0.05); nevertheless, the correlation between miRNA 145 and miRNA 155 was significant (r = 0.782, P = 0.008). Insignificant correlations were observed between age/gender and levels of the study miRNAs using the Spearman correlation and independent t-tests, respectively (P > 0.05).

**Figure 4:**
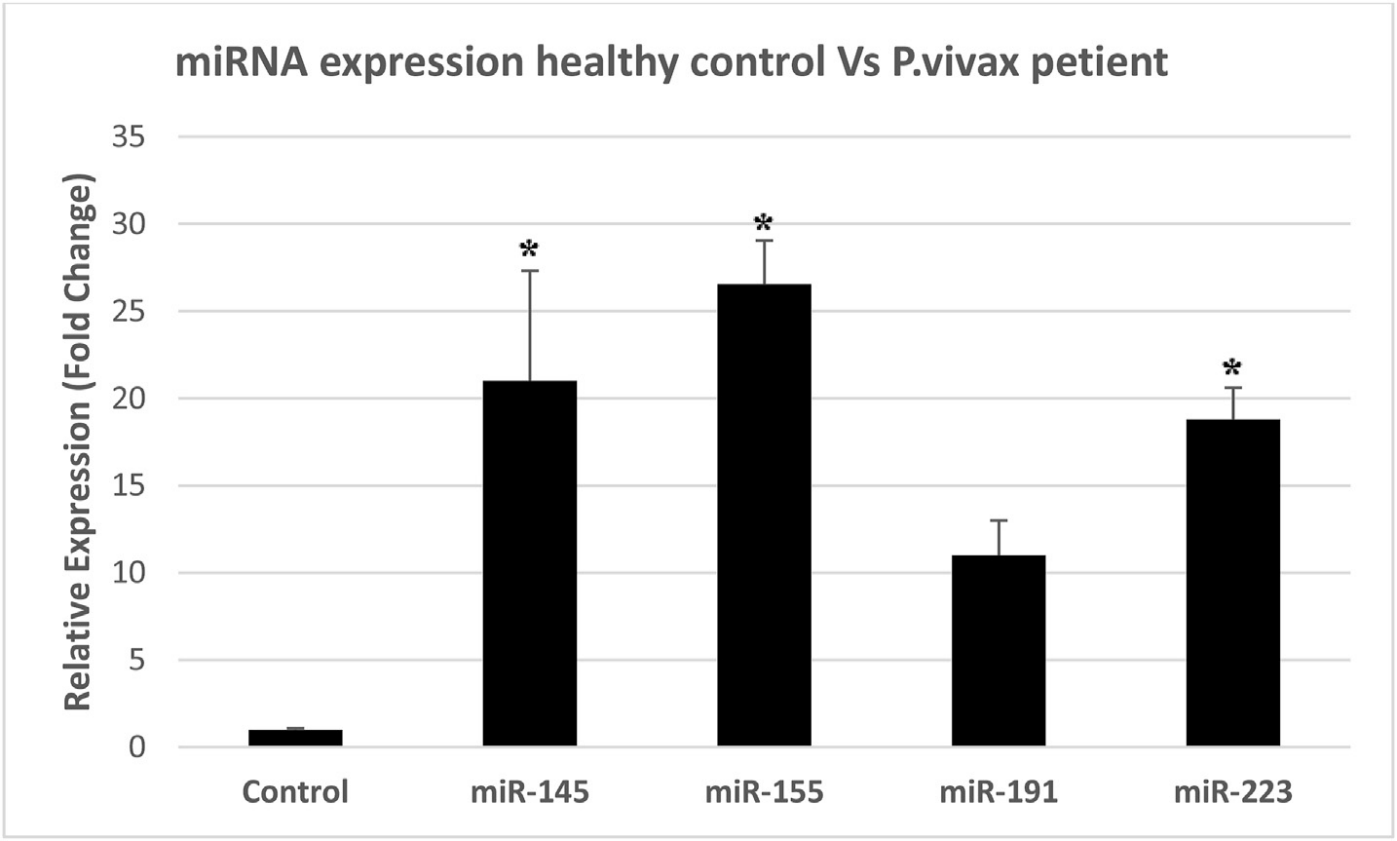
Real-Time qPCR expression analysis; expression of miR-145, miR-155, miR-191 and miR-223 was evaluated in plasma of patients with *P. vivax* in comparison with healthy controls. For the evaluation of the expression level of each miRNA was used the 2−ΔCt method. P < 0.05 was considered significant (∗ indicates P < 0.05).

**Figure 5:**
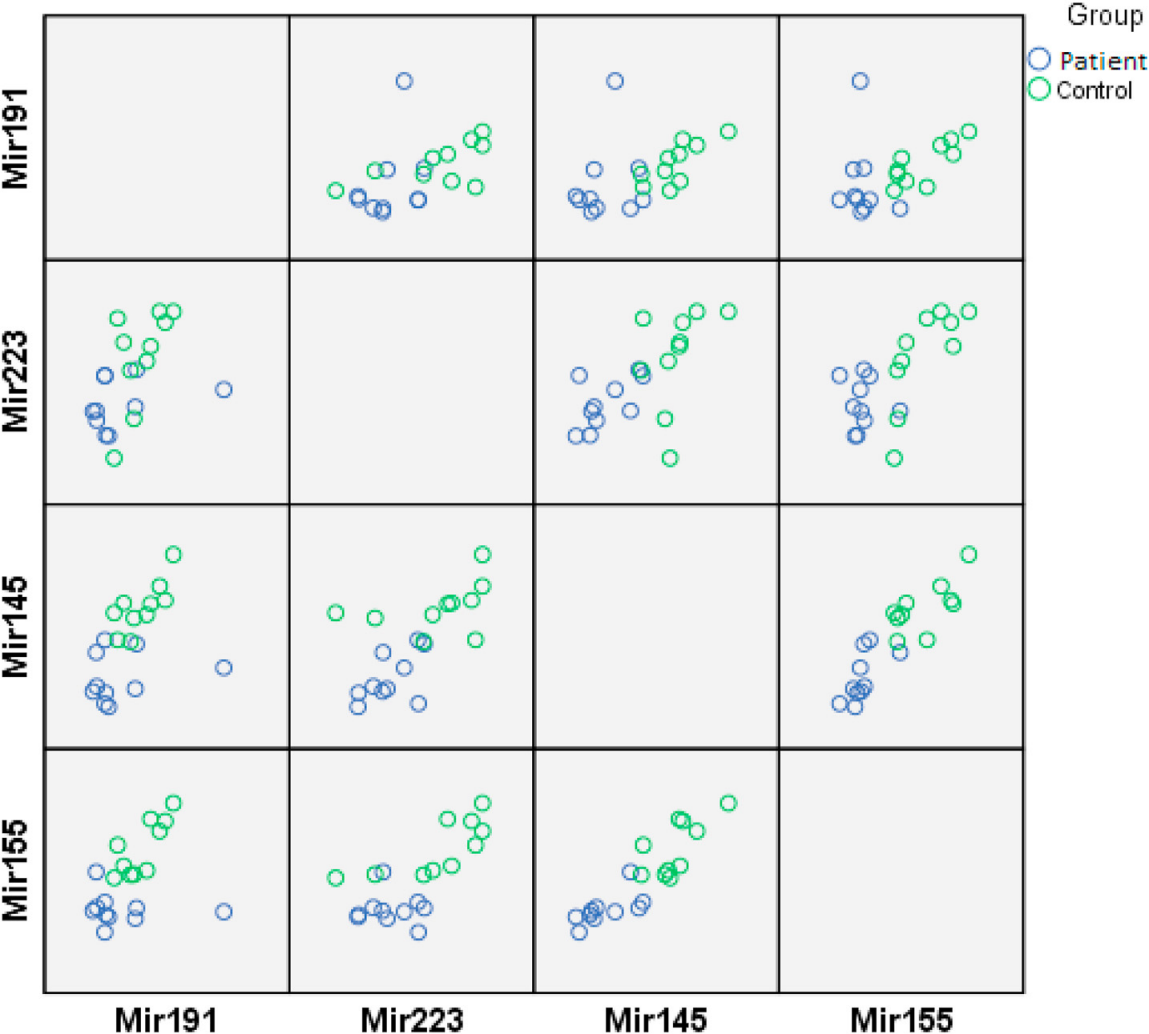
miRNA expression data for each patient and healthy control.

According to the Real-Time PCR analysis by 2−ΔCt method, the expressions of miR-223, miR-145 and miR-155 were respectively found to be 5.6, 16.9 and 1.7 times higher in patients with *P. vivax* compared to those in the healthy individuals (Figure 4). The expressions of all the three miRNAs significantly increased in patients with malaria compared to those in the controls (P < 0.05). Although the expression of miR-191 was 1.405 times higher in the patients with malaria compared to the controls, this difference was statistically insignificant. Figure 5 shows the miRNA expression as a scatter plot, which individually highlights the data of each patient and healthy control. Also, there is a significant difference at P < 0.05 in miRNAs expression between healthy controls and patients by Mann-Whitney U test (Table 6).

**Table 6:** Evaluation of miRNAs expression between healthy controls and patients by Mann-Whitney U test.

miRNA	U-value	Z-score	P-value
miR-191	20	−2.22999	0.02574
miR-223	18	−2.3	0.01732
miR-145	1	−3.66626	0.00024
miR-155	3	−3.51507	0.00044

## 4 Discussion

Changes in the expressions of miRNAs have been reported in many diseases, including viral, bacterial and parasitic infections [30, 31, 32, 33]. Research shows changes in the amount of miRNAs in infection with *P. vivax* in humans [23]. Infection with *Plasmoduim* changed certain miRNAs, including mir-16, mir-27, mir-150 and mir-451, in the plasma or cerebral and hepatic cells of rodents [18]. A number of miRNAs, including hsa-miR-7977, were upregulated in patients with complicated *P. vivax* malaria compared to in those with uncomplicated *P. vivax* malaria [34]. Anti-malaria medications such as chloroquine were found to be related to changes in miRNAs levels in patients [35]. Moreover, miRNAs can serve as appropriate markers for monitoring the pathophysiological status and diagnosing malaria. According to Gupta et al., microRNAs such as hsa-mir-4497 can be used for the early diagnosis of severe malaria caused by *P. falciparum*, which can help predict and more effectively treat this condition [16]. In addition, hsa-miR-3158-3p can be used as a biomarker for the prognosis of cerebral malaria caused by *P. falciparum* in children and adults [17].

In addition to high specificity and sensitivity, the accuracy, reliability and detection capability of biomarkers should be acceptable [36, 37]. These non-invasive biomarkers can be used to evaluate and monitor the pathophysiological status given that miRNAs secreted in biological fluids such as plasma can be detected after a long time and even after enduring harsh conditions. Efforts have been made to determine the associations of miRNAs in the serum, plasma, urine and other body fluids with different diseases, including malaria.

High serum levels of miR-223, miR-122 and miR-34a and low serum levels of miR-199a-3p, 199a-5p and miR-146b were reported in mice infected with *Schistosoma japonicum* [38]. Serum levels of miRNAs can be used as a biomarker for detecting infections in parasitic diseases.

In line with previous studies on infectious diseases, the present study evaluated miR-191, miR-223, miR-145 and miR-155 as miRNAs with significantly-high plasma levels in patients with *P. vivax* malaria. Differences were observed between patients with *P. vivax* malaria and healthy individuals in terms of the levels of miR-191, miR-223, miR-145 and miR-155.

Significant reductions in the mean plasma levels of miR-451 and miR-16 were reported in patients with *P. vivax* malaria [21]. There was also a weak negative correlation between the levels of parasitemia and our four study miRNAs. The highest correlation was observed between parasitemia and miR-233 (r = −0.23), but it was not statistically significant.

It appears that the increased level of parasitemia can cause downregulation of some miRNAs in plasma. Further studies are needed to better illustrate this point.

Some studies have shown that miRNAs cause pathological processes that are related to infectious diseases. When bacteria, viruses, parasites, or other pathogens enter an organism's body, hundreds of host genes are altered and miRNAs help eliminate the pathogens [31]. The present study found significant increases in the levels of miR-223, miR-145 and miR-155 compared to the control group (P < 0.05); nevertheless, the serum levels of miR-191 were insignificantly lower than those in the controls (P > 0.05). It is recommended that more comprehensive studies be conducted using larger samples to investigate the roles of these miRNAs.

Martin-Alonso et al. reported significant increases in the serum levels of miR-146a in plasmodium-infected mice compared to healthy mice [18]. They observed increases in a special type of miRNA in malaria, which is consistent with the present findings. They also found significant increases in the plasma miR-146a to regulate the immune function in malaria by producing inflammatory cytokines. These inflammatory cytokines eventually activate different immune mechanisms in the rat body [18]. Wang et al. examined miRNAs released from erythrocytes infected with *P. falciparum in vitro* [33]. They found the release of miR-451/140 from RBCs to decrease the expression of PFEMP1 as an essential parasite antigen and produce an intrinsic resistance to malaria in adult erythrocytes. Despite the differences between the present research and that by Wang in the type of miRNAs (RBC vs plasma) and *Plasmodium* species (*P. falciparum* vs *P. vivax)*, both studies found infection with malaria affects the expression of different miRNAs and that the species and strain of infectious agents affect the level of the microRNA expressions. More extensive research is required for confirming this finding.

Some studies suggest complications such as liver degradation, hemolysis and cerebral malaria are more common in *P. falciparum* and therefore intravascular hemolysis induces higher plasma levels of miRNAs in *P. falciparum* compared to *P. vivax* [39].

The plasma levels of miRNA are not always elevated in infectious diseases, as some studies have shown a slight or significant decrease in the plasma levels of miR-150 in patients with sepsis levels [30], miR-451 in patients with renal cell carcinoma (non-infectious) and miR-16 in patients with nasopharyngeal carcinoma (non-infectious) compared to the healthy control group [32]. The present study did not find decreases in the mirRNAs expression in patients with *P. vivax*.

The present study limitations comprised its small sample of patients with malaria. The implementation of a malaria elimination program has dramatically decreased the number of patients with malaria in Iran. In 2018, out of 631 cases observed in this country, 98.4% came mainly from Pakistan and Afghanistan [29].

Immediately isolating plasma upon sampling and storing it in a freezer were essential for examining miRNAs. The one-year sampling period was another limitation of the present research, which restricted the amount of plasma collected from patients with malaria.

Further studies are required for identifying more reliable biomarkers and clarifying their mechanism. The present study pioneered the investigation of the expression levels of miR-223, miR-145 and miR-155 in infection with malaria. These small molecules can serve as biomarkers for infection with *P. vivax*.

## 5 Conclusion

Despite the confirmed role of miRNAs as non-invasive biomarkers in acute human infectious diseases, the associations of these molecules with malaria pathogenesis are yet to be clarified. The present study found *P. vivax* to change host miRNAs such as miR-223, miR-145 and miR-155. These small molecules can therefore serve as biomarkers for risk assessment, disease detection, prognosis and monitoring the treatment of *P. vivax*.

## Declarations

### Author contribution statement

Ramtin Hadighi, Hossein Keshavarz: Conceived and designed the experiments.

Aliehsan Heidari, Parviz Fallah: Conceived and designed the experiments; Analyzed and interpreted the data; Contributed reagents, materials, analysis tools or data; Wrote the paper.

Ziba Tavakoli, sholeh Mansouri: Performed the experiments.

Mehrdokht Sadrkhanloo: Analyzed and interpreted the data; Wrote the paper.

### Funding statement

This work was supported by 10.13039/100012021Iran University of Medical Sciences (IUMS), Grant No. 31708.

### Data availability statement

Data included in article/supplementary material/referenced in article.

### Declaration of interests statement

The authors declare no conflict of interest.

### Additional information

No additional information is available for this paper.

## Appendix A Supplementary data

Supplementary Figure 1_V2.docx Supplementary Figure 1_V2.docx
