# Supplementary material for: Key plasma microRNAs variations in patients with Plasmodium vivax malaria in Iran
Source: Heliyon. 2022 Feb 27;8(3):e09018. doi: 10.1016/j.heliyon.2022.e09018 (PMC8899233; doi:10.1016/j.heliyon.2022.e09018)

Supplementary Figure 1: Malaria - Reference pathway; Pasmodium protozoa are parasites that account for malaria infection. Sporozoite forms of the parasite are injected by mosquito bites under the skin and are carried to the liver where they develop into the merozoite form. Sporozoite invasion of hepatocytes is mediated by parasite surface protein like CSP. Subsequent infection into red blood cells (RBCs) by merozoites causes malaria disease via aberrant cytokine production and sequestration of parasite-infected red blood cells (pRBCs) to host endothelium. Microvasculature sequestration in the brain brings about cerebral malaria that can results in death or persisting neurological impairment. PfEMP1 has been suggested as the key adhesive molecule of pRBCs.


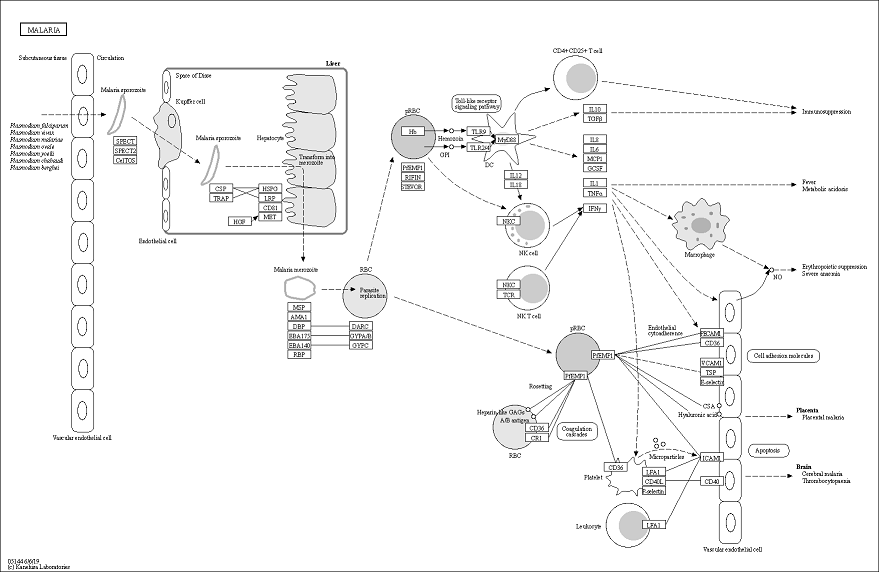

Supplement: Supplementary Figure 1_V2.docx [file mmc1.docx]
